# Automated machine learning model to predict anti-tuberculosis drug-induced liver injury in patients with tuberculous meningitis

**DOI:** 10.3389/fphar.2026.1816387

**Published:** 2026-08-12

**Authors:** Pengyu Li, Yang Yang, Lanyan Xi, Ling Li, Ying Zeng, Yanmei Mao, Pan Yan

**Affiliations:** 1 Department of Pharmacy, The Affiliated Changsha Central Hospital, Hengyang Medical School, University of South China, Changsha, China; 2 College of Pharmacy, Changsha Medical University, Changsha, China

**Keywords:** anti-tuberculosis drug-induced liver injury, automated machine learning, gradient boosting machine, predictive model, tuberculous meningitis

## Abstract

**Background:**

Tuberculous meningitis (TBM) is a severe central nervous system infection with high disability and mortality rates. However, during TBM treatment, anti-tuberculosis drug-induced liver injury (ATB-DILI) often precipitates treatment interruption and contributes to poor clinical outcomes. This study aims to develop an automatic machine learning (AutoML) model for predicting the risk of ATB-DILI in TBM patients.

**Methods:**

A retrospective cohort study was conducted in adult TBM patients. After feature selection via least absolute shrinkage and selection operator (LASSO) regression, AutoML was employed to construct predictive models. The bootstrap resampling method was used for internal validation. Furthermore, feature importance, partial dependence plots, and SHapley Additive exPlanations (SHAP) analysis were utilized for model interpretation.

**Results:**

A total of 253 TBM patients were included in this study, of whom 54 (21.34%) developed ATB-DILI. LASSO regression analysis identified four characteristic factors, including cerebrospinal fluid (CSF) chloride, blood platelet, hypertension, and total bilirubin. Among the candidate models generated by AutoML, the optimal gradient boosting machine (GBM) model demonstrated superior performance. It achieved an optimism-corrected area under the receiver operating characteristic curve (AUC) of 0.828 (95% CI: 0.794–0.861) and an optimism-corrected area under the precision-recall curve (PR-AUC) of 0.711 (95% CI: 0.628–0.782). In addition, interpretability analysis revealed that CSF chloride was the most important variable for the optimal GBM model.

**Conclusion:**

The ATB-DILI prediction model, developed using AutoML technology, demonstrated high predictive ability and interpretability. It can assist clinicians in identifying TBM patients at risk of ATB-DILI, thereby optimizing patient management and facilitating the formulation of personalized medication regimens.

## 1 Introduction

Tuberculous meningitis (TBM), a central nervous system infection caused by *Mycobacterium tuberculosis* (*M. tuberculosis*), is the most severe manifestation of tuberculosis with an exceptionally high disability and mortality rate (Shi et al., 2023). A 2025 meta-analysis found that the all-cause mortality rate of TBM patients is up to 27.7% and 41.7% of survivors exhibited long-term neurological impairment (Afazel et al., 2025). Currently, more than 100,000 new TBM cases are estimated to occur per year, presenting a major challenge for health systems, economy and society (Huynh et al., 2022; Lourens et al., 2025). In 2020, the World Health Organisation (WHO) launched the first global road map to defeat meningitis by 2030, highlighting the urgent need to improve global surveillance and reduce the burden of meningitis (World Health Organization, 2021).

The recommended standard regimen for drug-sensitive TBM is derived from the treatment protocols of pulmonary tuberculosis, consisting of an intensive phase for at least 2 months followed by a consolidation phase (Huynh et al., 2022). However, given the severity of the disease and poor drug penetration, a longer treatment duration, typically 9–12 months, is advised for TBM (Donovan et al., 2025). As reported, anti-tuberculosis drug-induced liver injury (ATB-DILI) is the most common adverse reaction leading to treatment interruption, while discontinuation of anti-tuberculosis drugs has been identified as an independent risk factor for death from TBM (Donovan et al., 2025). More alarmingly, previous research has demonstrated a higher incidence of ATB-DILI in TBM patients compared to those with pulmonary tuberculosis, probably driven by the high-dose drug demands, unavoidable polypharmacy, and the elevated physiological stress inherent to meningitis (Kumar et al., 2017; Ruslami et al., 2022; Semwal et al., 2025). In view of the significant impact of ATB-DILI on the treatment course and prognosis of TBM patients, it is particularly important to identify and implement early preventive measures for high-risk patients.

In recent years, machine learning (ML) technology has been widely used in the medical field. ML can effectively handle and analyze large volumes of diverse data sources, including electronic health records (EHRs), genomic data, and clinical trial reports, for the enhanced detection and prediction of adverse drug reactions and toxicity (Yang and Kar, 2023; Sun et al., 2026). Automated machine learning (AutoML), an emerging field in machine learning, can intelligently develop high-quality models tailored to the target data through automated algorithm selection and hyperparameter tuning (Zhang et al., 2024). Its efficiency and user-friendliness allow cross-disciplinary experts without specialist knowledge of artificial intelligence to benefit from these advances (Ma et al., 2024). In our previous study, AutoML was employed to predict the risk of liver injury in pediatric tuberculosis treatment, and the optimal model demonstrated excellent performance (Zeng et al., 2025). However, no study has used ML or AutoML to identify the ATB-DILI in TBM patients.

In this study, AutoML technique was applied for the first time to predict the risk of ATB-DILI in TBM patients. This novel AutoML-based risk prediction tool is expected to rapidly and accurately assess the risk of ATB-DILI in TBM patients, thereby facilitating personalized therapy and improving the prognosis.

## 2 Methods

### 2.1 Study design and subjects

In this study, patients diagnosed with TBM and treated with the standard anti-tuberculosis regimen were enrolled from the Affiliated Changsha Central Hospital of University of South China between January 2019 and June 2026. According to case definition criteria, TBM was categorized into definite TBM and probable/possible TBM based on symptoms and signs of meningitis, along with the diagnostic score. The anti-tuberculosis regimen consists of first-line anti-tuberculosis drugs [isoniazid + rifampicin + pyrazinamide (or + ethambutol)] for the intensification period.

**FIGURE 1:**
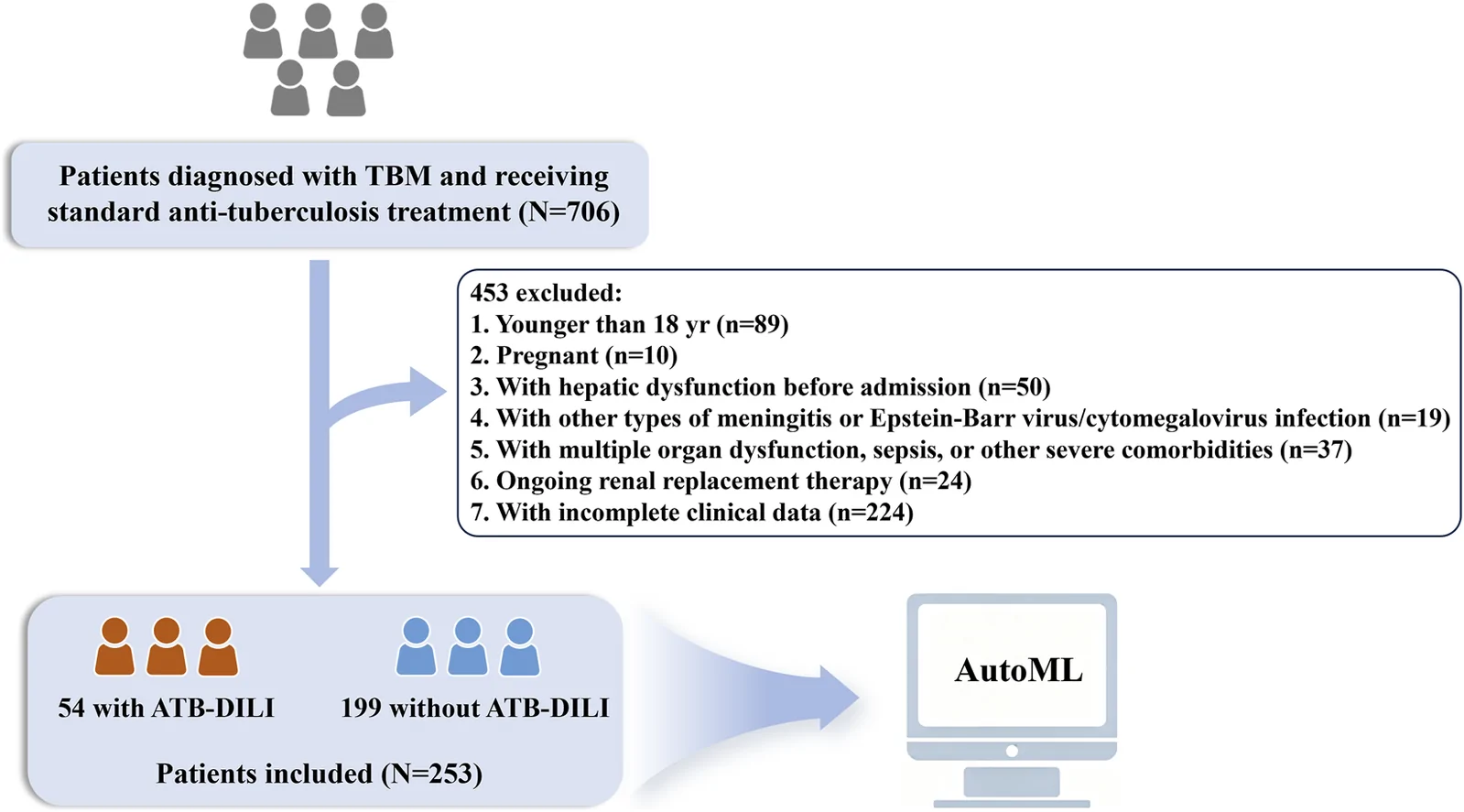
Flow chart of the study.

Exclusion criteria: (1) aged <18 years; (2) pregnant; (3) had liver disease or hepatic dysfunction before admission; (4) combined with other types of meningitis, Epstein-Barr virus or cytomegalovirus infection; (5) had multiple organ dysfunction, sepsis, or other severe comorbidities; (6) ongoing renal replacement therapy; (7) with incomplete clinical data. From an initial pool of 706 patients, exclusions were made for those meeting any of the above exclusion criteria, resulting in a final sample of 253 eligible patients for analysis (Figure 1).

### 2.2 Diagnosis of ATB-DILI

All enrolled patients presented with normal liver biochemistry at baseline, and their liver function were monitored for 2 months following treatment initiation. The diagnostic of ATB-DILI was established based on guidelines published by the Chinese Medical Association and literature (Wang et al., 2022; Ji et al., 2023; Chinese Medical Association Tuberculosis Branch, 2024). The diagnostic criteria were defined as follows: a) alanine aminotransferase (ALT) exceeding threefold upper limit of normal (ULN) and/or total bilirubin (TBil) exceeding twofold ULN; b) aspartate aminotransferase (AST), alkaline phosphatase (ALP) and total bilirubin elevated at the same time, and at least one of them exceeding twofold ULN. A severity grading scale of DILI is shown in Supplementary Table S1 (Xiao et al., 2019).

### 2.3 Data collection

Baseline clinical data were collected from the electronic medical record system (EMRS) within 72 h prior to initiation of TBM therapy. Information on the demographics, clinical manifestations, comorbidities, concomitant medications and dosages of anti-tuberculosis drugs were also documented for all patients.

### 2.4 Feature selection

Feature selection was performed on the entire dataset using the least absolute shrinkage and selection operator (LASSO) algorithm, combined with 5-repeated stratified 10-fold cross-validation. LASSO streamlines the architecture of subsequent modeling by eliminating features with unstable contributions, thereby alleviating overfitting and improving interpretability (Sahu and Fatma, 2025; Wu et al., 2025). Specifically, 10-fold cross-validation was selected to ensure a more comprehensive evaluation of the model’s performance across different data subsets (Yu et al., 2024). Based on lambda.min, variables exhibiting non-zero coefficients in over 50% of the LASSO cross-validation trials were selected as the final predictive factors (Sullivan et al., 2025). In addition, the variance inflation factor (VIF) was calculated to assess multicollinearity among the selected variables.

### 2.5 Model development and validation

To maximize data utilization, the entire dataset was employed to develop the final AutoML predictive models. The prediction model was developed using the AutoML technology of the ‘H_2_O’ R package (www.h2o.ai) (LeDell and Poirier, 2020). The search space of AutoML models included distributed random forest (DRF), generalized linear models (GLM), gradient boosting machines (GBM), extremely randomized trees (XRT), deep neural networks (DNN), stacked ensembles and XGBoost. In this study, hyperparameter optimization was performed using the RandomDiscrete search strategy, which randomly sampled hyperparameter combinations to efficiently identify optimal model parameters. In H_2_O AutoML, the loss function is automatically specified by the distribution parameter. For the Bernoulli distribution, the corresponding loss function is logloss (Candel et al., 2016). To optimize computational efficiency and ensure sufficient validation samples, a 5-fold cross-validation was implemented (Yu et al., 2024), and then a leaderboard was created to display the candidate models and their performance metrics. These metrics included the area under the receiver operating characteristic curve (AUC), logloss, area under the precision-recall curve (PR-AUC), and mean_per_class_error. Given the relatively limited sample size, traditional data partitioning and cross-validation techniques may fail to provide stable and reliable validation estimates (Peng et al., 2024). Therefore, bootstrap resampling (n = 1,000 iterations) was employed for internal validation. By repeatedly random sampling with replacement from the original cohort to create multiple subsets for model validation, this approach reduces validation variance and yields more robust performance estimates than fixed data splits, particularly for small samples (Wen et al., 2024). Crucially, this approach allowed us to quantify the overfitting bias and derive the strict optimism-corrected performance estimates reported in our study.

As a key technique for handling class imbalance, threshold moving can significantly improve the performance of classifiers (Amiri et al., 2025). The H_2_O AutoML platform facilitates the selection of an appropriate threshold based on metrics such as the maximum F1, maximum F2, accuracy, or precision. After determining the optimal model, we selected the decision threshold that maximized the F2 score to enhance sensitivity and minimize the risk of missed ATB-DILI cases (Ikemura et al., 2021; Zeng et al., 2025).

In the study, the H_2_O open-source package (version 3.46.0.11) was installed via R (version 4.5.1), and then implemented by RStudio (version 2025.09.1). The apparent and optimism-corrected calibration curves were constructed using localized regression smoothing (LOESS) via the standard stats package and visualized using the ‘ggplot2’ R package. Additionally, the decision curve analysis (DCA) was performed using the ‘dcurves’ R package. Additionally, a logistic regression model was developed for comparison with the AutoML model. DeLong’s test was employed to compare AUC values.

### 2.6 Model explainability

The model explainability was evaluated through feature importance, partial dependence, SHapley Additive exPlanation (SHAP), and local interpretable model-agnostic explanations (LIME) plots.

### 2.7 Statistical analysis

Multiple imputation was performed to handle missing data prior to conducting statistical analyses. Categorical variables were analyzed using the chi-squared (χ^2^) test or Fisher’s exact test, and continuous variables were compared using the *t*-test or the nonparametric Mann-Whitney U test. A *p*-value <0.05 was considered statistically significant. All statistical analyses were performed using GraphPad Prism 9 (GraphPad Software, Inc., San Diego, CA, United States).

## 3 Results

### 3.1 Demographic and clinical characteristics

A total of 253 TBM patients were enrolled in this study. The mean age of patients was 48.73 ± 18.53 years, with males accounting for 63.64%. Common presenting symptoms included fever (63.24%), headache (47.04%), infarction (28.46%), and altered consciousness (18.97%). Of the enrolled patients, 54 cases (21.34%) developed ATB-DILI following the anti-tuberculosis therapy, including 47 cases classified as grade 1 (mild liver injury) and 7 cases classified as grade 2 (moderate liver injury), respectively. This study collected 40 potential variables from demographic characteristics, clinical data and medication. Missing values, restricted exclusively to CSF ADA (n = 2, 0.79%), were imputed via Multivariate Imputation by Chained Equations (MICE) with Predictive Mean Matching (PMM). Detailed imputation parameters and descriptive diagnostics are summarized in Supplementary Tables S2, S3, respectively. The results of univariate analysis are displayed in Table 1. Compared to the No-DILI group, the DILI group had a higher proportion of patients with hypertension, as well as significantly higher levels of age, total bilirubin, alanine aminotransferase, neutrophil percentage, CSF leucocyte, CSF protein and CSF ADA. Conversely, patients in the DILI group exhibited significantly lower levels of blood platelet count, serum sodium, and CSF chloride.

**TABLE 1:** Comparison of the characteristics of patients using univariate analysis.

Characteristics	Total (n = 253)	DILI (n = 54)	No-DILI (n = 199)	*P*-value
Age (years), median (IQR)	51 (30–64)	59 (41–70)	49 (29–62)	0.0238^a^
Sex (male/female)	161/92	32/22	129/70	0.4509^b^
Body weight (kg), median (IQR)	55.00 (49.00–62.50)	54.00 (47.75–64.00)	55.00 (50.00–61.50)	0.7356^a^
History of alcohol, n (%)	43 (17.00)	12 (22.22)	31 (15.58)	0.2490^b^
Clinical signs and symptoms, n (%)				
Altered consciousness	48 (18.97)	15 (27.78)	33 (16.58)	0.0628^b^
Infarction	72 (28.46)	21 (38.89)	51 (25.63)	0.0554^b^
Hydrocephalus	19 (7.51)	7 (12.96)	12 (6.03)	0.1397^c^
Fever	160 (63.24)	40 (74.07)	120 (60.30)	0.0627^b^
Headache	119 (47.04)	27 (50.00)	92 (46.23)	0.6226^b^
Comorbidities, n (%)				
Diabetes	39 (15.42)	8 (14.81)	31 (15.58)	0.8905^b^
Hypertension	51 (20.16)	21 (38.89)	30 (15.08)	0.0001^b^
Coadministration of drugs, n (%)				
Combination with PPI	171 (67.59)	39 (72.22)	132 (66.33)	0.4121^b^
Combination with corticosteroids	204 (80.63)	46 (85.19)	158 (79.40)	0.3398^b^
Combination with linezolid	76 (30.04)	20 (37.04)	56 (28.14)	0.2060^b^
Combination with quinolones	182 (71.94)	35 (64.81)	147 (73.87)	0.1891^b^
Laboratory investigations				
Albumin (g/L), mean ± SD	33.36 ± 4.79	32.69 ± 4.71	33.55 ± 4.81	0.2412
Total bilirubin (μmol/L), median (IQR)	8.70 (6.30–13.11)	11.38 (7.89–17.88)	8.14 (6.10–12.17)	0.0017^a^
Direct bilirubin (μmol/L), median (IQR)	3.50 (2.30–5.41)	3.95 (2.70–6.35)	3.45 (2.30–5.01)	0.0602^a^
Alanine aminotransferase (U/L), median (IQR)	16.00 (10.00–25.50)	20.50 (14.75–27.75)	14.00 (9.00–25.00)	0.0040^a^
Aspartate aminotransferase (U/L), median (IQR)	22.00 (16.00–30.00)	23.50 (18.00–30.00)	21.00 (15.00–30.00)	0.1163^a^
Total biliary acid (μmol/L), median (IQR)	4.20 (2.65–7.11)	4.34 (2.70–7.73)	4.20 (2.60–7.00)	0.8372^a^
Creatinine (μmol/L), median (IQR)	55.00 (44.00–68.50)	52.00 (41.75–65.00)	55.00 (44.00–71.00)	0.3466^a^
Urea nitrogen (mmol/L), median (IQR)	4.22 (3.34–5.76)	4.14 (3.28–5.80)	4.22 (3.37–5.76)	0.9929^a^
White blood cell (10^9^/L), median (IQR)	6.46 (5.25–8.85)	6.98 (5.32–9.32)	6.41 (5.15–8.74)	0.4536^a^
Red blood cell (10^9^/L), mean ± SD	4.01 ± 0.68	3.90 ± 0.65	4.04 ± 0.69	0.1722
Neutrophil (10^9^/L), median (IQR)	5.14 (3.82–7.13)	5.82 (3.72–8.06)	5.00 (3.83–7.00)	0.2572^a^
Neutrophil percentage (%), median (IQR)	79.30 (72.85–85.00)	81.70 (76.68–87.35)	78.40 (71.70–84.40)	0.0159^a^
Hemoglobin (g/L), mean ± SD	112.45 ± 19.31	113.54 ± 19.76	112.16 ± 19.23	0.6432
Blood platelet (10^9^/L), mean ± SD	260.54 ± 92.45	230.02 ± 80.78	268.81 ± 93.86	0.0060
ESR (mm/h), median (IQR)	34.00 (19.50–59.50)	30.50 (18.25–51.50)	36.00 (20.00–62.00)	0.1361^a^
CRP (mg/L), median (IQR)	16.60 (5.10–42.40)	19.09 (5.12–44.53)	15.80 (5.01–41.30)	0.6655^a^
Serum sodium (mmol/L), median (IQR)	136.00 (131.00–139.00)	134.00 (128.00–136.25)	136.00 (132.00–139.00)	0.0048^a^
Serum potassium (mmol/L), mean ± SD	3.92 ± 0.44	3.91 ± 0.49	3.93 ± 0.43	0.7504
CSF leucocyte (10^6^/L), median (IQR)	52.00 (6.00–191.00)	136.50 (22.50–286.25)	36.00 (5.00–181.00)	0.0032^a^
CSF protein (g/L), median (IQR)	1.01 (0.50–1.79)	1.32 (0.83–2.48)	0.91 (0.46–1.71)	0.0027^a^
CSF chloride (mmol/L), median (IQR)	120.00 (113.00–125.50)	115 (104.75–121.00)	121.00 (113.00–127.00)	<0.0001^a^
CSF ADA (U/L), median (IQR)	3.60 (1.00–6.60)	5.70 (3.00–9.20)	3.00 (0.80–6.00)	0.0002^a^
Daily dose of drugs per weight				
Isoniazid dose (mg/kg/d), median (IQR)	9.09 (7.69–10.48)	8.92 (7.80–10.09)	9.09 (7.69–10.55)	0.9064^a^
Rifampicin dose (mg/kg/d), median (IQR)	9.23 (8.18–10.33)	9.00 (7.50–10.00)	9.23 (8.18–10.47)	0.1031^a^
Pyrazinamide dose (mg/kg/d), mean ± SD	26.36 ± 5.61	26.27 ± 5.22	26.70 ± 6.88	0.6140

Abbreviations: IQR, interquartile range; SD, standard deviation; PPI, proton pump inhibitor; CRP, C-reactive protein; ESR, erythrocyte sedimentation rate; CSF, cerebrospinal fluid; ADA, adenosine deaminase.

^a^ Mann-Whitney U-test.

^b^ Chi-squared test.

^c^ Fisher’s exact test.

### 3.2 Factor identification

**FIGURE 2:**
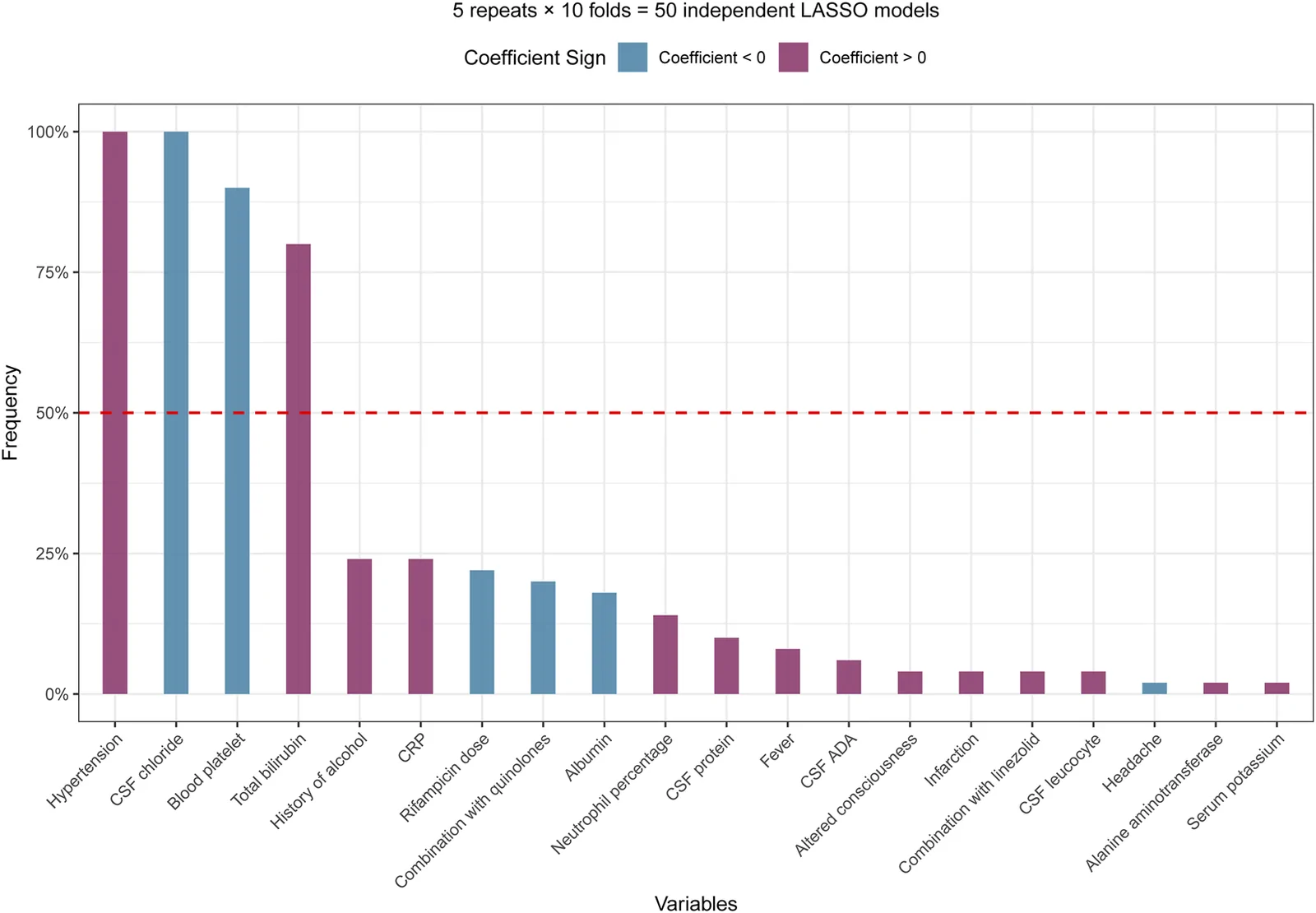
Frequency of non-zero coefficients in 5-repeated stratified 10-fold cross-validation for variable selection using LASSO regression.

Forty variables were analyzed using the LASSO algorithm to discern the characteristic factors. As shown in Figure 2, four variables were selected for model development, including hypertension, total bilirubin, blood platelet, and CSF chloride. Furthermore, all VIF values among the selected variables were less than 2, indicating no significant multicollinearity.

### 3.3 Development and validation of predictive model

**FIGURE 3:**
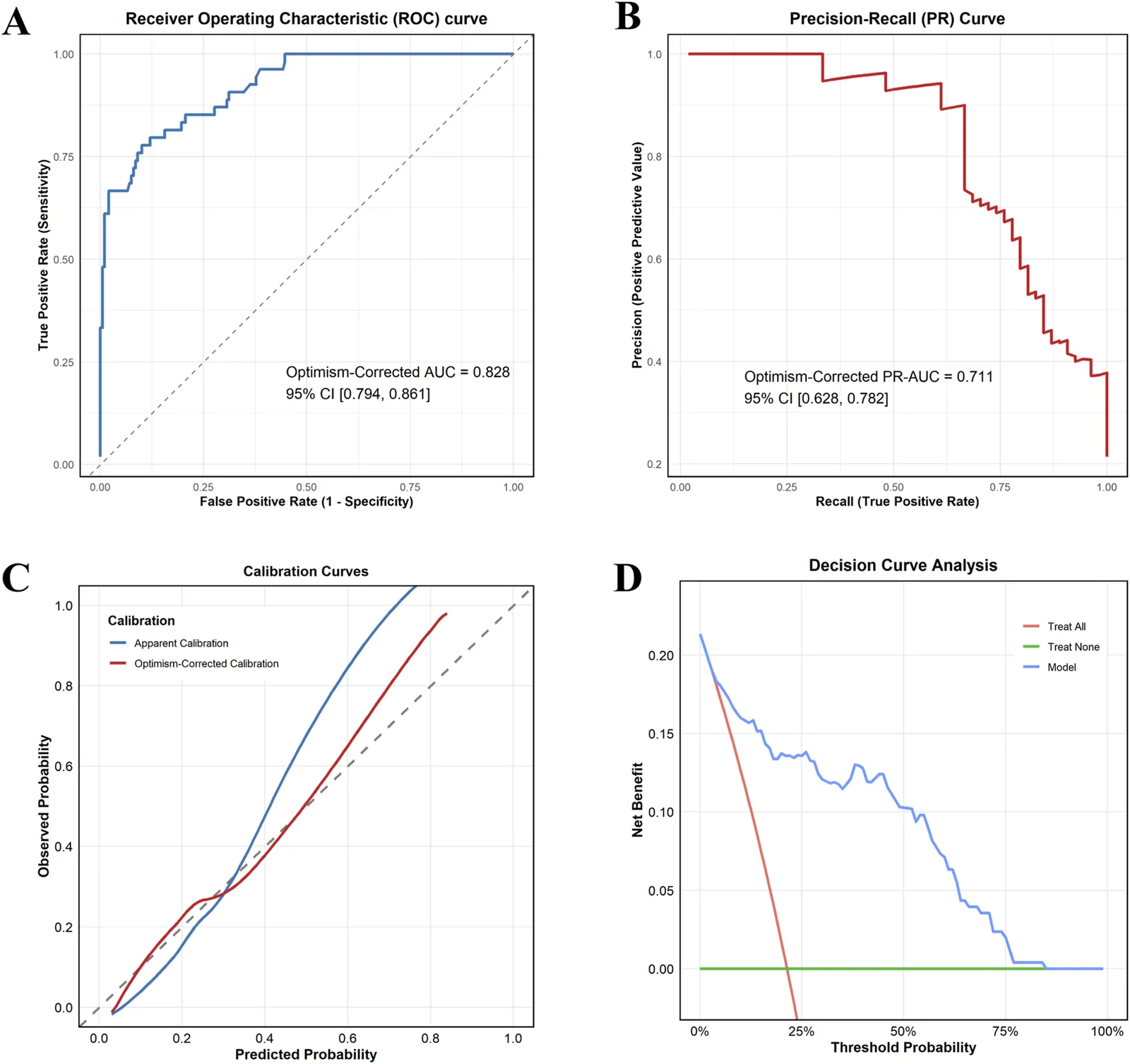
Comprehensive performance evaluation of the optimal GBM model. **(A)** Receiver operating characteristic (ROC) curve with optimism-corrected AUC and 95% CI (based on 1,000 bootstrap resamples); **(B)** Precision-recall (PR) curve with optimism-corrected PR-AUC and 95% CI (based on 1,000 bootstrap resamples); **(C)** Calibration curves; **(D)** Decision curve analysis.

Using AutoML technology, numerous machine learning models were constructed. The top 20 candidate models, as determined by cross-validation performance, are presented in Table 2. Furthermore, it was found that the top position on the leaderboard was occupied by the GBM model, which exhibited the highest AUC score and the second-lowest logloss value. This indicates superior performance in both classification accuracy and probabilistic prediction quality. The optimal GBM model (Model_ID: GBM_grid_1_AutoML_2_20260711_155949_model_3) was selected for further evaluation and in-depth analysis. Its hyperparameters are shown in Supplementary Table S4. The apparent AUC and PR-AUC of the optimal GBM model were 0.921 and 0.828, respectively. Based on bootstrap internal validation (n = 1,000), the optimal GBM model achieved an optimism-corrected AUC of 0.828 (95% CI: 0.794–0.861) and an optimism-corrected PR-AUC of 0.711 (95% CI: 0.628–0.782) (Figures 3A,B). It exhibited good calibration and excellent clinical net benefit (Figures 3C,D). Furthermore, DeLong’s test demonstrated that the AUC of the optimal GBM model was significantly higher than that of the standard logistic regression model (*p* < 0.01; Supplementary Figure S1). The decision threshold was determined by maximizing the F2-score. At this threshold, the precision, sensitivity, specificity, accuracy, F1-score and F2-score of the optimal GBM model are presented in Supplementary Table S5.

**TABLE 2:** The leaderboard of machine learning models ranked by 5-fold cross-validated performance.

Model_id	AUC	logloss	RR-AUC	mean_per_class_error
GBM_grid_1_AutoML_2_20260711_155949_model_3	0.749	0.458	0.400	0.283
StackedEnsemble_BestOfFamily_1_AutoML_2_20260711_155949	0.741	0.462	0.402	0.310
DeepLearning_grid_1_AutoML_2_20260711_155949_model_1	0.735	0.533	0.429	0.302
GLM_1_AutoML_2_20260711_155949	0.735	0.455	0.446	0.323
DeepLearning_grid_1_AutoML_2_20260711_155949_model_2	0.735	0.491	0.411	0.313
GBM_4_AutoML_2_20260711_155949	0.725	0.472	0.387	0.319
StackedEnsemble_AllModels_1_AutoML_2_20260711_155949	0.723	0.475	0.386	0.320
DeepLearning_grid_2_AutoML_2_20260711_155949_model_2	0.716	0.467	0.392	0.326
DeepLearning_grid_3_AutoML_2_20260711_155949_model_2	0.711	0.466	0.399	0.332
GBM_grid_1_AutoML_2_20260711_155949_model_5	0.710	0.469	0.385	0.344
GBM_3_AutoML_2_20260711_155949	0.706	0.484	0.355	0.337
DeepLearning_grid_3_AutoML_2_20260711_155949_model_1	0.702	0.513	0.414	0.333
DeepLearning_1_AutoML_2_20260711_155949	0.701	0.512	0.373	0.317
GBM_2_AutoML_2_20260711_155949	0.687	0.493	0.331	0.341
GBM_grid_1_AutoML_2_20260711_155949_model_4	0.670	0.570	0.318	0.349
DeepLearning_grid_2_AutoML_2_20260711_155949_model_1	0.664	0.568	0.417	0.356
GBM_grid_1_AutoML_2_20260711_155949_model_1	0.662	0.616	0.294	0.324
GBM_5_AutoML_2_20260711_155949	0.654	0.547	0.281	0.349
DRF_1_AutoML_2_20260711_155949	0.646	0.708	0.299	0.373
XRT_1_AutoML_2_20260711_155949	0.645	0.692	0.271	0.359

Models of the same algorithm type but with different names correspond to distinct hyperparameter settings. Abbreviations: AUC, area under the curve; logloss, logarithmic loss; mean_per_class_error, mean per-class error; GBM, gradient boost machine; GLM, generalized linear models; XRT, extremely randomized trees; DRF, distributed random forest.

### 3.4 Model interpretation

**FIGURE 4:**
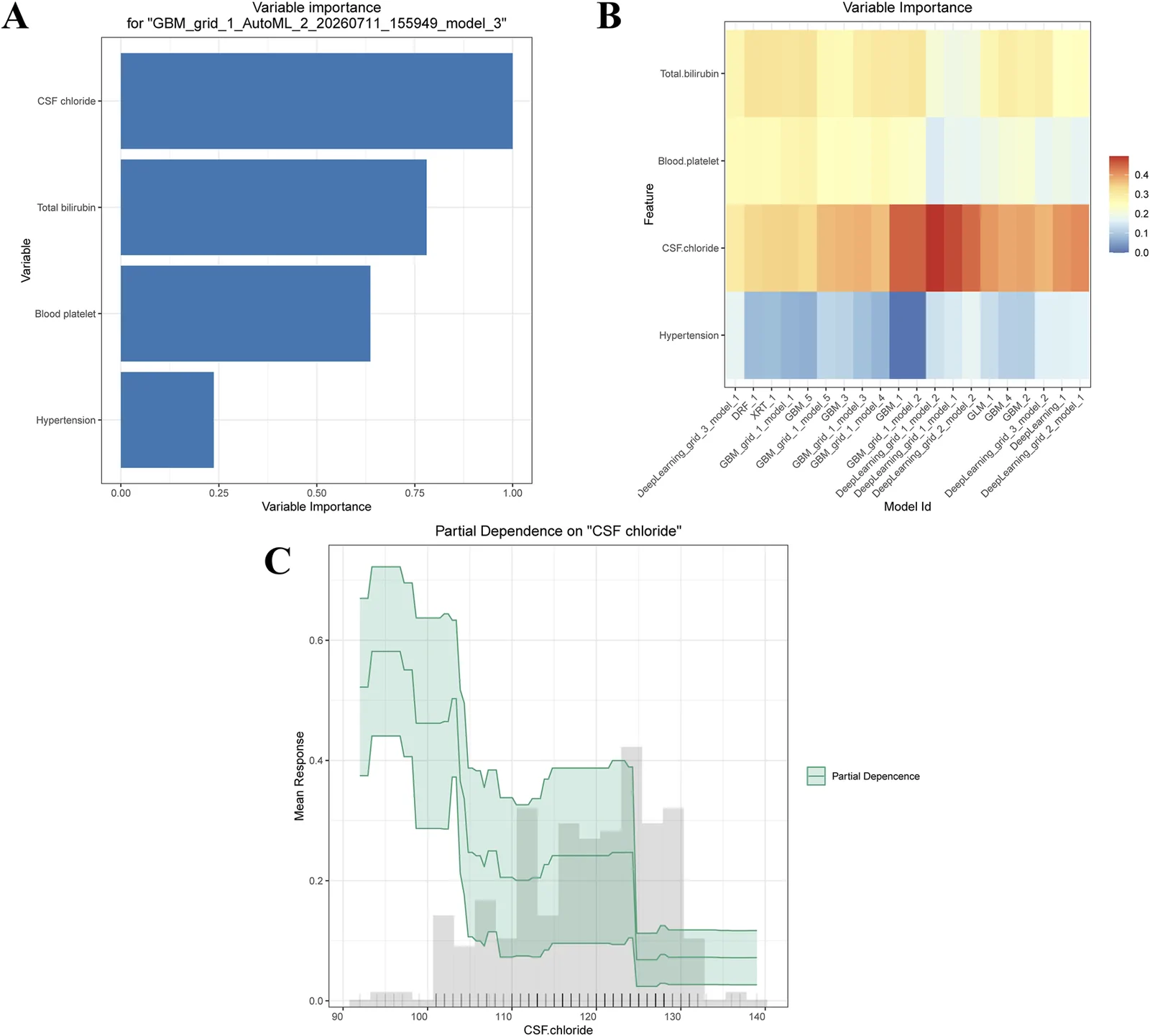
Variable importance and partial dependence analyses. **(A)** Variable importance scores for the optimal GBM model; **(B)** Heatmap of variable importance across multiple models; **(C)** Partial dependence plot for CSF chloride levels in the optimal GBM model.

As shown in Figure 4A, the variable importance plot for the optimal GBM model indicated that CSF chloride was the pivotal variable, followed by total bilirubin, blood platelet, and hypertension. Furthermore, a variable importance heatmap was employed to visualize the significance of features in different models. These results consistently identified CSF chloride as the most crucial feature for predictive models (Figure 4B). The partial dependence plot illustrates the marginal effect of a target feature on the predictions of an ML model (Liang et al., 2023). As shown in Figure 4C, this plot demonstrates that lower CSF chloride levels may be associated with a higher response of the optimal GBM model.

**FIGURE 5:**
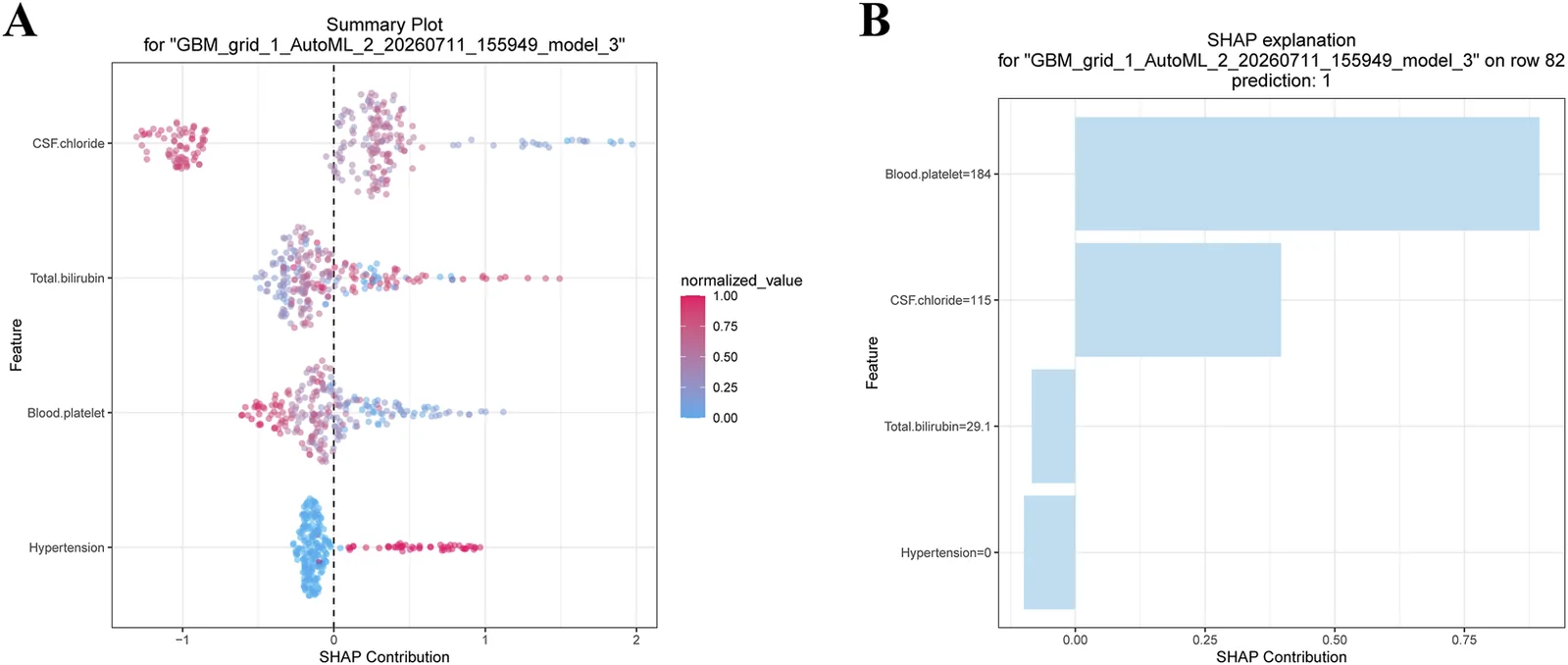
SHAP explanation of the optimal GBM model. **(A)** SHAP summary plot; **(B)** LIME plot for Patient #82.

SHAP analysis not only identifies the key features that drive model predictions and quantifies their global importance, but also explains the direction and magnitude of each feature’s effect on individual predictions, greatly enhancing model interpretability (Liu et al., 2023; Zhang et al., 2024). The SHAP plot generated by the optimal GBM model is illustrated in Figure 5A. The blue dots for CSF chloride and blood platelet count are largely clustered to the right of the zero line. This indicates that lower values of these two features are associated with a higher likelihood of ATB-DILI in TBM patients. LIME analysis can quantify the contribution of each feature to an individual prediction (Liu et al., 2023). For example, Figure 5B illustrates how each feature contributes positively or negatively to predicting ATB-DILI for Patient #82. For this patient, features such as blood platelet and CSF chloride showed relatively higher SHAP values, suggesting they were major contributors to the model’s prediction. Conversely, hypertension and total bilirubin had lower weights, indicating a minimal impact on the risk prediction in this specific case. The partial dependence plots for this patient are presented in Supplementary Figure S2.

## 4 Discussion

TBM is one of the most severe forms of tuberculosis, requiring long-term standardized treatment. The occurrence of ATB-DILI can lead to treatment interruption, and thus cause a poor outcome. The incidence rate of ATB-DILI varies widely across countries, with 9.5%–14.1% reported in China (Jiang et al., 2021; Li et al., 2023). By comparison, TBM patients enrolled in our study exhibited a higher incidence rate of ATB-DILI (21.34%). Previous studies have indicated that ATB-DILI is more common among patients with high disease burden, such as disseminated tuberculosis and TBM (Kumar et al., 2017; Misra et al., 2021). Consistent with this observation, a recent meta-analysis involving 38,971 patients demonstrated that extrapulmonary tuberculosis constitutes a significant risk factor for ATB-DILI (XIE et al., 2024). The underlying causes of the increased risk of ATB-DILI in TBM patients have not been fully elucidated but are hypothesized to be related to higher drug doses, polypharmacy, and physiological stress. Owing to the existence of the blood-brain barrier, high-dose anti-tuberculosis drugs are typically required to attain sufficient therapeutic concentrations in the cerebrospinal fluid, potentially raising the risk of dose-dependent ATB-DILI (Ruslami et al., 2022). Furthermore, the clinical management of TBM often necessitates adjunctive therapies, including corticosteroids, anti-edema agents, and antiepileptic drugs, some of which possess hepatotoxic potential that may exacerbate liver injury (Semwal et al., 2025). In addition to drug-related factors, host determinants also contribute to the development of DILI. TBM patients commonly exhibit heightened physiological stress and systemic inflammation, a pathophysiological state known to predispose to hepatic dysfunction (Misra et al., 2020; Misra et al., 2021). Given the substantial impact of ATB-DILI on the management and prognosis of TBM, it is critical to establish a TBM-specific predictive model that enables clinicians to perform focused monitoring and initiate timely interventions for high-risk patients. In this study, the AutoML was employed to develop an optimal predictive model for ATB-DILI in TBM patients.

LASSO analysis revealed four key predictors, including hypertension, total bilirubin, platelet count and CSF chloride. Growing evidence suggests that hypertension increases the risk of ATB-DILI (Xu et al., 2022; Nyangwara et al., 2025). Patients with hypertension often require long-term antihypertensive therapy. Antihypertensive agents including calcium channel blockers, angiotensin-converting enzyme inhibitors, and angiotensin receptor blockers carry inherent hepatotoxic potential and may therefore exacerbate liver injury when co-administered with anti-tuberculosis drugs (Park et al., 2021). Total bilirubin, a critical indicator of liver function, reflects basal hepatic metabolic capacity and is a well-recognized risk factor for DILI development (Zhang et al., 2022; Ji et al., 2023). Elevated bilirubin level may indicate underlying hepatic metabolic disturbance, predisposing patients to ATB-DILI upon initiation of anti-tuberculosis treatment (Liu et al., 2024; Zeng et al., 2025). Platelet production is primarily regulated by thrombopoietin (TPO) from the liver. Beyond their role in hemostasis, platelets have been shown to promote hepatic regeneration and protect against cholestatic liver injury (Lambert, 2016; Lisman and Luyendyk, 2018). Sometimes, reduced platelet counts may imply compromised hepatic detoxification and adaptive capacity.

In addition to conventional biochemical markers, TBM-related CSF parameters were also investigated in the study. TBM patients typically presents with elevated CSF leucocyte count, protein, adenosine deaminase and reduced CSF chloride compared to healthy individuals (Zou et al., 2015; Ye and Yan, 2023; Donovan et al., 2025). Accordingly, patients in our cohort showed abnormal CSF parameters relative to normal range, with more pronounced alterations observed in those with ATB-DILI (Table 1). Accumulating evidence links these CSF alterations to physiological stress and inflammatory responses driven by *M. tuberculosis* infection (Kumar et al., 2017; Kalita et al., 2019; Cresswell et al., 2021). We hypothesize that, CSF chloride, an important feature identified in our study, serves as a surrogate marker for TBM severity, disease stage and systemic inflammatory burden. These disease states may heighten hepatic vulnerability to hepatotoxic agents (Villanueva-Paz et al., 2021; Lim et al., 2023). However, the biological mechanisms underlying the association between TBM-specific CSF parameters and ATB-DILI remain to be fully elucidated in future investigations.

ML is increasingly employed to predict adverse drug reactions by analyzing high-dimensional data, including electronic health records, drug pharmacodynamics, patient-specific biomarkers, and treatment history (Yahya et al., 2025). Currently, multiple studies have employed ML for the early detection of hepatotoxicity and have demonstrated high predictive accuracy. For example, one study developed several machine learning models to predict the risk of hepatotoxicity in patients receiving nilotinib, with AUC values ranging from 0.61 to 0.65 (Kim et al., 2021). Another study developed an individualized nomogram based on a logistic regression model to predict DILI risk in tuberculosis patients during anti-tuberculosis treatment, with reported AUCs of 0.833, 0.668, and 0.753 in the training group, validation group I and validation group II, respectively (Ji et al., 2023). Furthermore, a risk scoring system for hepatotoxicity within 1 year of tyrosine kinase inhibitor initiation was developed using ML methods (including multivariate logistic regression, elastic net, and random forest), which achieved an AUC of 0.755 (Han et al., 2022). In this study, we developed and validated a set of models using AutoML technology to predict the risk of ATB-DILI in TBM patients, among which the optimal GBM model demonstrated strong discriminative performance with an optimism-corrected AUC of 0.828 (95% CI: 0.794–0.861) and an optimism-corrected PR-AUC of 0.711 (95% CI: 0.628–0.782). Compared to conventional logistic regression, our model developed using AutoML demonstrates higher predictive performance and requires only minimal manual parameter tuning. This paradigm lowers the barrier to entry for non-ML experts, enabling them to build high-quality predictive models efficiently. Additionally, the SHAP method was employed to interpret the optimal GBM model. This enhancement in clinical interpretability enables clinicians to understand the model’s rationale, thereby facilitating personalized treatment planning.

This study has several limitations. First, while our institution is the primary government-designated tuberculosis center with good regional representativeness, the overall sample size was constrained by the relative rarity of TBM and strict inclusion and exclusion criteria. To address this limitation, we performed rigorous internal validation using 1,000 bootstrap resamples instead of a conventional single train-test split, and reported optimism-corrected performance metrics (including AUC and PR-AUC). Nonetheless, statistical adjustment cannot fully compensate for limited sample size. Large-scale, multi-center prospective studies are thus required to externally validate our optimized GBM model and improve its clinical applicability. Second, despite the availability of clinical genetic testing at our center, genetic variables related to anti-tuberculosis drug metabolism were excluded from model construction due to extensive missing data. Integration of these pharmacogenetic variables in future studies may further enhance the model’s predictive performance.

## 5 Conclusion

In summary, this study is the first application of AutoML to develop a prediction model for ATB-DILI in TBM patients. CSF chloride, blood platelet, hypertension, and total bilirubin were identified as characteristic factors. Among the candidate models, the optimal GBM model demonstrated superior performance in predicting ATB-DILI, with an optimism-corrected AUC of 0.828 (95% CI: 0.794–0.861) and an optimism-corrected PR-AUC of 0.711 (95% CI: 0.628–0.782). This predictive model facilitates early risk assessment of ATB-DILI in TBM patients prior to anti-tuberculosis treatment, allowing for timely intervention to improve survival and clinical outcomes.

## Data Availability

The original contributions presented in the study are included in the article/Supplementary Material, further inquiries can be directed to the corresponding authors.
