# Supplementary material for: Automated machine learning model to predict anti-tuberculosis drug-induced liver injury in patients with tuberculous meningitis
Source: Front Pharmacol. 2026 Aug 12;17:1816387. doi: 10.3389/fphar.2026.1816387 (PMC13506474; doi:10.3389/fphar.2026.1816387)
Supplement: Supplementary file 1 [file Supplementaryfile1.docx]

**Figure S1.** Receiver operating characteristic (ROC) curve of the logistic regression model.

**Figure S2**. Partial dependence plots for Patient #82 from the optimal GBM model. (A) Blood platelet; (B) CSF chloride; (C) Total bilirubin; (D) Hypertension.

**Table S1.** Grading of DILI severity.

| **Grade** | **Description** | **Definition** |
| --- | --- | --- |
| 0 | No liver injury | Patient can tolerate the exposed drug without hepatotoxic reaction. |
| 1 | Mild | Serum ALT and/or ALP show a reversible increase, with TBil < 2.5×ULN and INR<1.5 |
| 2 | Moderate | Serum ALT and/or ALP are increased, and TBil≥2.5×ULN or INR≥1.5 |
| 3 | Severe | Serum ALT and/or ALP are increased, TBil≥5×ULN, with or without INR≥1.5 |
| 4 | Acute | Serum ALT and/or ALP are increased and TBil≥10×ULN, or a daily increase of ≥1.0 mg/dL (17.1 μmol/L), and INR≥2.0 or PTA<40%. |
| 5 | Lethal injury | Death due to DILI or the need for liver transplantation to survive |

Abbreviations: DILI: drug-induced liver injury; ALT, alanine aminotransferase; ALP, alkaline phosphatase; TBil, total bilirubin; ULN, upper limit of normal; INR, international normalized ratio; PTA, prothrombin activity.

**Table S2.** Detailed overview of missing data handling and imputation strategy for the initial dataset.

|  | Missing Count (n) | Missingness (%) | Assumed Mechanism | Imputation Framework | Specific Imputation Model | Number of Imputations |
| --- | --- | --- | --- | --- | --- | --- |
| CSF ADA | 2 | 0.79 | MCAR^a^ | MICE | PMM | 5 |
| All other candidate predictors | 0 | 0 | NA | NA | NA | NA |
| Outcome Variable | 0 | 0 | NA | NA | NA | NA |

Abbreviations: CSF, Cerebrospinal fluid; ADA, adenosine deaminase; MCAR, Missing Completely at Random; MICE, Multivariate Imputation by Chained Equations; PMM, Predictive Mean Matching

Note: a, Little’s MCAR test

**Table S3.** The descriptive diagnostics of multiple interpolation results

| **Data** | **Imputation** | **N** | **Mean** | **Std. Deviation** | **Minimum** | **Maximum** |
| --- | --- | --- | --- | --- | --- | --- |
| **Original Data** |  | 251 | 4.4312 | 4.30536 | .0000 | 26.0000 |
| **Imputed Values** | 1 | 2 | .5000 | .00000 | .5000 | .5000 |
|  | 2 | 2 | .9500 | .07071 | .9000 | 1.0000 |
|  | 3 | 2 | 1.8500 | 1.62635 | .7000 | 3.0000 |
|  | 4 | 2 | 1.1000 | .14142 | 1.0000 | 1.2000 |
|  | 5 | 2 | .9000 | .42426 | .6000 | 1.2000 |
| **Complete Data After Imputation** | 1 | 253 | 4.4001 | 4.30240 | .0000 | 26.0000 |
|  | 2 | 253 | 4.4036 | 4.29935 | .0000 | 26.0000 |
|  | 3 | 253 | 4.4108 | 4.29557 | .0000 | 26.0000 |
|  | 4 | 253 | 4.4048 | 4.29842 | .0000 | 26.0000 |
|  | 5 | 253 | 4.4032 | 4.29975 | .0000 | 26.0000 |

**Table S4.** Hyperparameters of the optimal GBM model in H2O AutoML.

| **Hyperparameter** | **Final_Value** |
| --- | --- |
| ntrees | 29 |
| max_depth | 3 |
| min_rows | 1 |
| learn_rate | 0.1 |
| sample_rate | 0.7 |
| col_sample_rate | 0.7 |
| col_sample_rate_per_tree | 0.7 |
| min_split_improvement | 1e-05 |
| histogram_type | UniformAdaptive |
| categorical_encoding | Enum |
| distribution | bernoulli |
| nfolds | 5 |
| seed | 7 |
| stopping_metric | logloss |
| stopping_tolerance | 0.05 |
| calibration_method | PlattScaling |
| fold_assignment | Modulo |
| score_tree_interval | 5 |

**Table S5.** The performance metrics of the optimal GBM model.

| **Sensitivity** | **Specificity** | **Precision** | **Accuracy** | **F1-score** | **F2-score** |
| --- | --- | --- | --- | --- | --- |
| 0.7963 | 0.8794 | 0.6418 | 0.8617 | 0.7107 | 0.7597 |

Note: threshold = 0.2471
